# Supplementary material for: Postcopulatory sexual selection and the evolution of shape complexity in the carnivoran baculum
Source: Proc Biol Sci. 2020 Oct 14;287(1936):20201883. doi: 10.1098/rspb.2020.1883 (PMC7657853; doi:10.1098/rspb.2020.1883)
Supplement: Sensitivity analyses [file rspb20201883supp4.docx]

**Supplementary Material S4**

Data were reanalysed having removed all felid species from the dataset, to explore the effect of the complex (yet potentially ‘residual’) cat baculum on our results. All trends reported in the main body of the MS remain present when felids are removed.


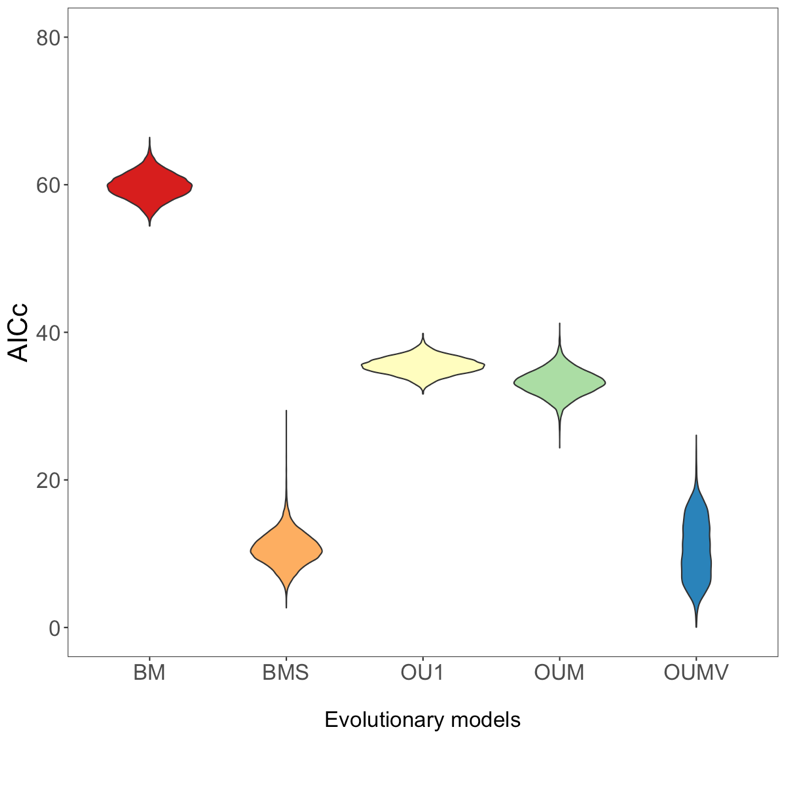


**Modified Figure 3.** (minus cats)

| **Model** | **AICc** | **ΔAICc** | **AICcW** |
| --- | --- | --- | --- |
| BM | 59.8 | 49.18 | 0.00 |
| **BMS** | **10.6** | **0.00** | **0.51** |
| OU1 | 35.5 | 24.85 | 0.00 |
| OUM | 33.2 | 22.56 | 0.00 |

| **OUMV** | **10.7** | **0.08** | **0.49** |
| --- | --- | --- | --- |

**Modified Table 1.** (minus cats)


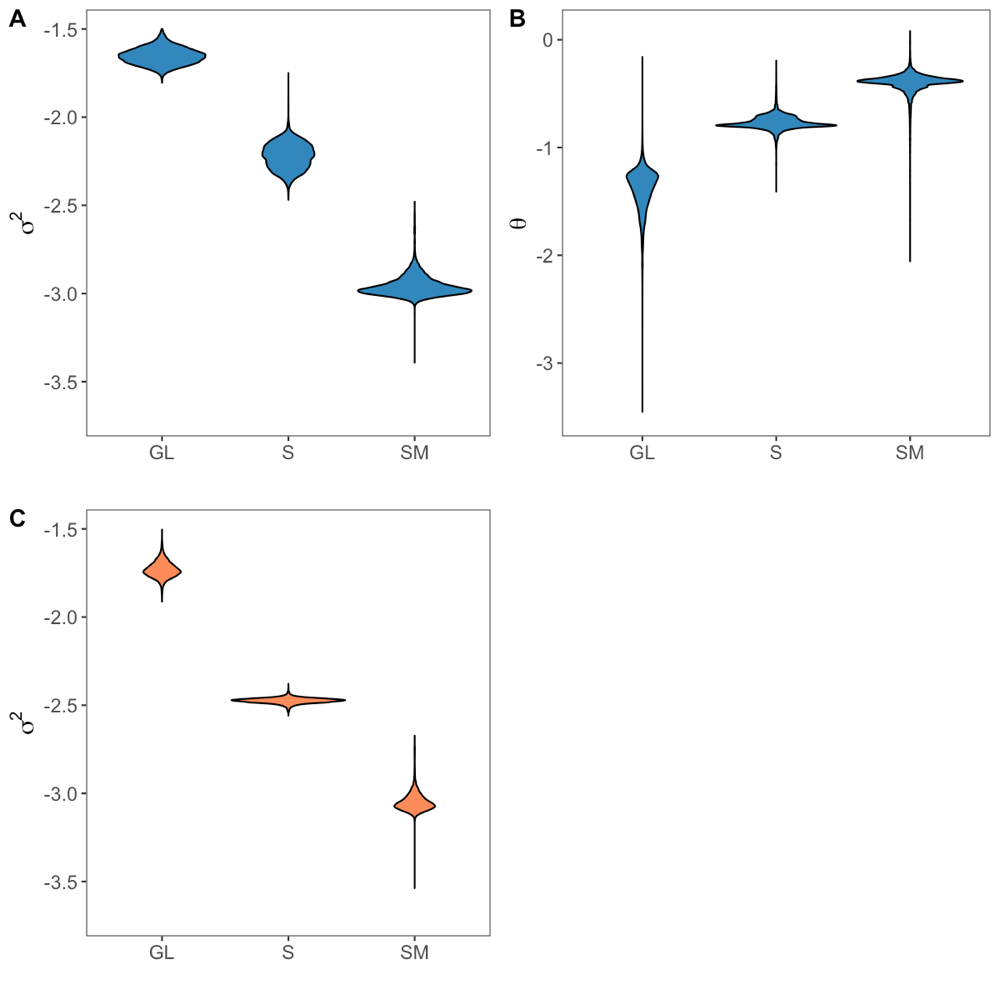


**Modified Figure 4.** (minus cats)

| **Trait** | **n** | **λ** | **α ± SE** | ***r*^2^** | **Predictor** | **β ± SE** | ***p*** | **β CI** |
| --- | --- | --- | --- | --- | --- | --- | --- | --- |
| Baculum length | 47 | 0.85 | 1.33±0.12 | 0.61 | Testes mass | 0.16±0.08 | 0.028* | 0.03 - 0.29 |
|  |  |  |  |  | Body mass | 0.22±0.08 | 0.003** | 0.09 - 0.35 |
| Baculum complexity | 47 | 0.95 | -0.58±0.24 | 0.15 | Testes mass | 0.11±0.13 | 0.181 | -0.10 – 0.33 |
|  |  |  |  |  | Body mass | -0.31±0.12 | 0.009** | -0.52 - -0.10 |

**Modified Table 2.** (minus cats)

**Correlated Evolution** (minus cats):

Baculum length ~ relative testes size, logBF = -0.14

Baculum complexity ~ relative testes, logBG = -0.15

**Regional ANOVA** (minus cats):

Within-subject ANOVA = significant regional variation (ε=0.80, *F*_27.6, 305.4_, *p*=<0.001).

No different tip-base (*p*=0.77)

But significantly different tip-mid and mid-base (*p*<0.001)

| **Trait** | **n** | **λ** | **α ± SE** | ***r*^2^** | **Predictor** | **β ± SE** | ***p*** | **β CI** |
| --- | --- | --- | --- | --- | --- | --- | --- | --- |
| Baculum complexity |  |  |  |  |  |  |  |  |
| (whole) | 47 | 0.76 | -0.83±0.24 | <0.01 | Intromission | 0.05±0.10 | 0.319 | -0.13-0.23 |
| (tip) | 47 | 0.89 | -1.17±0.29 | 0.06 | Intromission | 0.18±0.11 | 0.053• | -0.01-0.36 |
| (midshaft) | 47 | 0.82 | -1.05±0.38 | 0.02 | Intromission | -0.14±0.16 | 0.817 | -0.40-0.12 |
| (base) | 47 | 0.87 | -0.84±0.30 | <0.01 | Intromission | 0.06±0.11 | 0.311 | -0.13-0.25 |
| Baculum complexity |  |  |  |  |  |  |  |  |
| (whole) | 64 | 0.87 | -0.91±0.24 | 0.02 | Ovulation | 0.19±0.15 | 0.112 | -0.07-0.44 |
| (tip) | 64 | 0.89 | -1.18±0.28 | 0.04 | Ovulation | 0.31±0.18 | 0.046* | 0.01-0.61 |
| (midshaft) | 64 | 0.87 | -1.41±0.39 | 0.01 | Ovulation | 0.22±0.26 | 0.200 | -0.21-0.66 |
| (base) | 64 | 0.80 | -0.86±0.28 | <0.01 | Ovulation | 0.07±0.20 | 0.354 | -0.26-0.40 |

**Regional BayesTraits regressions** (minus cats)

The inclusion of some (n=5) testes mass from the literature that *included* the associated epididymis likely introduced some degree of noise into the analysis. BayesTraits analyses were therefore rerun excluding these individuals, but the patterns remained

| **Trait** | **n** | **λ** | **α ± SE** | ***r*^2^** | **Predictor** | **β ± SE** | ***p*** | **β CI** |
| --- | --- | --- | --- | --- | --- | --- | --- | --- |
| Baculum length | 50 | 0.91 | 1.32±0.15 | 0.41 | Testes mass | 0.19±0.10 | 0.032* | 0.02 - 0.36 |
|  |  |  |  |  | Body mass | 0.11±0.09 | 0.126 | 0.02 - 0.30 |
| Baculum complexity | 50 | 0.94 | -0.62±0.24 | 0.04 | Testes mass | 0.09±0.15 | 0.268 | -0.16 – 0.34 |
|  |  |  |  |  | Body mass | -0.18±0.14 | 0.098 | -0.41 - 0.05 |

**Modified Table 2.** (minus testes with epididymis)

**Correlated Evolution** (minus testes with epididymis)

Baculum length ~ relative testes size, logBF = -0.32

Baculum complexity ~ relative testes, logBG = 0.58
