# Supplementary material for: Postcopulatory sexual selection and the evolution of shape complexity in the carnivoran baculum
Source: Proc Biol Sci. 2020 Oct 14;287(1936):20201883. doi: 10.1098/rspb.2020.1883 (PMC7657853; doi:10.1098/rspb.2020.1883)
Supplement: PGLS analysis of regional baculum complexity [file rspb20201883supp5.docx]

**Supplementary Material S2**

**Posterior distribution histograms of PGLS multiple regression parameters.** Analyses conducted in BayesTraits. A-D, relationship between baculum length and testes mass, with body mass as a covariate. E-H, relationship between baculum complexity and testes mass, with body mass as a covariate. Slope β_1_, regression coefficient for covariate (body mass); slope β_2,_ regression coefficient for independent variable (testes mass).

**
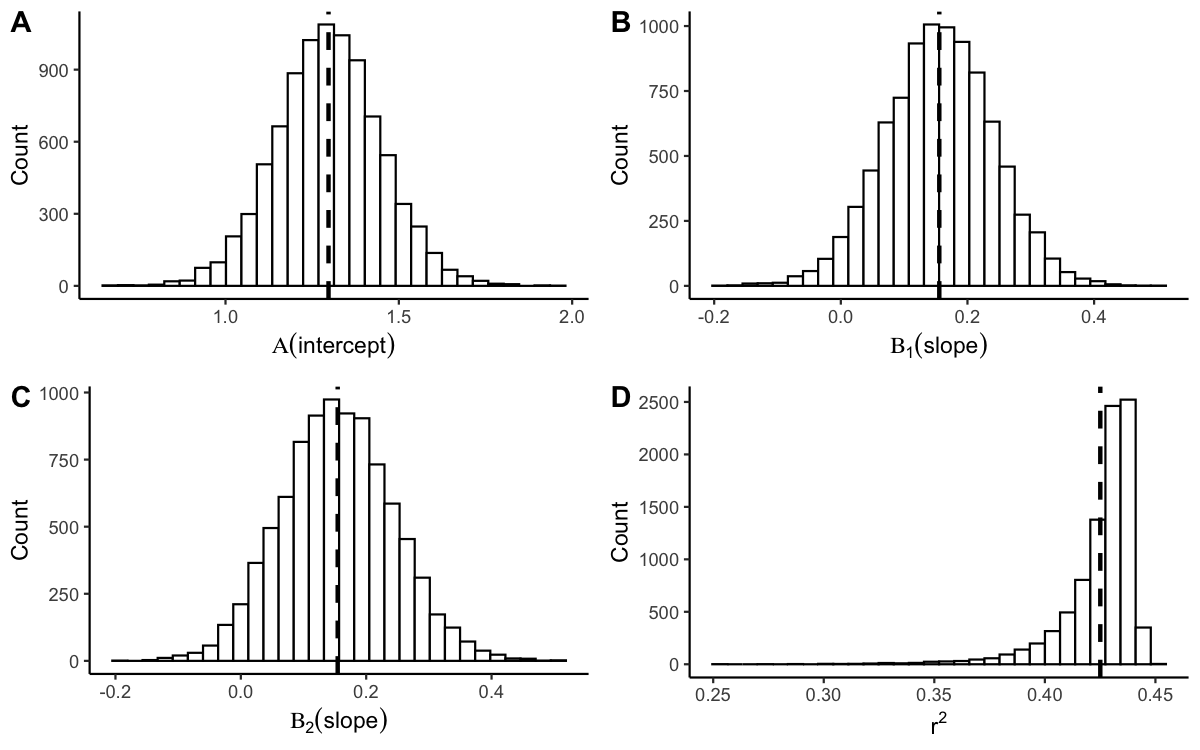
**

**
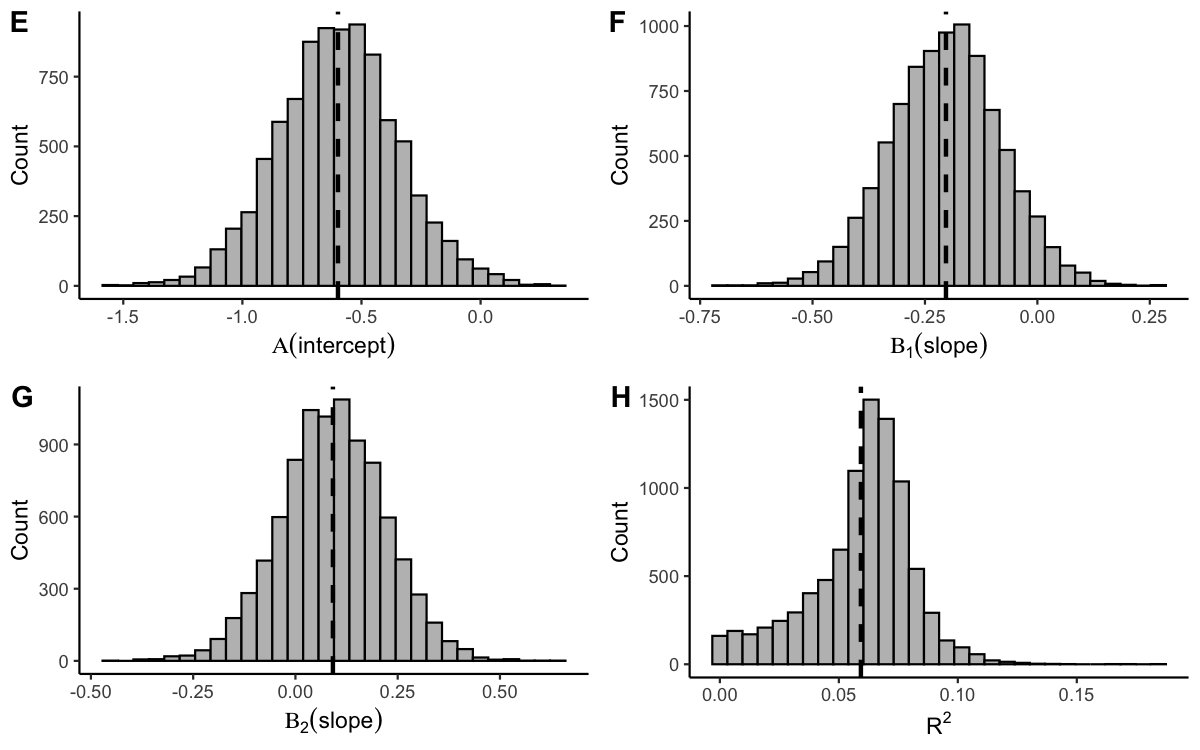
**

PGLS analysis of regional baculum complexity against reproductive parameters (intromission period and ovulation strategy)

| **Trait** | **n** | **λ** | **α ± SE** | ***r*^2^** | **Predictor** | **β ± SE** | ***p*** | **β CI** |
| --- | --- | --- | --- | --- | --- | --- | --- | --- |
| Baculum complexity |  |  |  |  |  |  |  |  |
| (whole) | 54 | 0.80 | -0.75±0.22 | <0.01 | Intromission | 0.04±0.08 | 0.318 | -0.10-0.18 |
| (tip) | 54 | 0.83 | -1.18±0.27 | 0.11 | Intromission | 0.24±0.10 | 0.020* | 0.05-0.43 |
| (midshaft) | 54 | 0.82 | -1.05±0.35 | 0.01 | Intromission | -0.09±0.13 | 0.768 | -0.31-0.12 |
| (base) | 54 | 0.86 | -0.71±0.27 | <0.01 | Intromission | -0.01±0.09 | 0.528 | -0.16-0.15 |
| Baculum complexity |  |  |  |  |  |  |  |  |
| (whole) | 72 | 0.87 | -0.85±0.22 | 0.02 | Ovulation | 0.20±0.15 | 0.091 | -0.04-0.45 |
| (tip) | 72 | 0.68 | -1.11±0.24 | 0.04 | Ovulation | 0.33±0.18 | 0.039* | 0.02-0.64 |
| (midshaft) | 72 | 0.88 | -1.32±0.38 | 0.01 | Ovulation | 0.25±0.26 | 0.170 | -0.18-0.67 |
| (base) | 72 | 0.80 | -0.80±0.26 | <0.01 | Ovulation | 0.09±0.19 | 0.315 | -0.23-0.40 |
